# Dental care for drug users in Norway: dental professionals’ attitudes to treatment and experiences with interprofessional collaboration

**DOI:** 10.1186/s12903-020-01240-1

**Published:** 2020-10-31

**Authors:** Ewa S. Hovden, Vibeke E. Ansteinsson, Ingrid Volden Klepaker, Eeva Widström, Rasa Skudutyte-Rysstad

**Affiliations:** 1 Oral Health Centre of Expertise in Eastern Norway (OHCE), Oslo, Norway; 2 Institute of Clinical Dentistry, Arctic University of Norway, Oslo, Norway

**Keywords:** Dental health services, Dental care, Drug users, Dental professionals’ attitudes, Interprofessional collaboration

## Abstract

**Background:**

The present study aimed to assess dental professionals’ attitudes and experiences related to the dental treatment of drug users and to interprofessional collaboration with the rehabilitation institutions (RIs).

**Methods:**

The study population comprised 141 dentists and dental hygienists (response rate 73%) working in the Public Dental Service (PDS) in three counties in Norway. All of the participants completed an electronically distributed questionnaire on existing practices and experiences regarding dental treatment for drug users and interprofessional collaboration with RIs. The Norwegian Centre for Research Data (NSD) approved the study.

**Results:**

Thirty-five percent of the dentists and 10% of the dental hygienists had treated five or more drug users per month (*p* <  0.05). The majority of dentists and dental hygienists used more time for examination and treatment of drug users compared to other patient groups. Over 70% of dental clinicians considered drug users as demanding patients due to fear, missing appointments, and poor compliance with oral hygiene advice. Multivariable logistic regression analyses showed that attitudes and experiences with dental treatment of drug users were significantly associated with background characteristics of professionals. The overall perception was that drug users often had higher expectations of dental treatment than what could be defined as necessary care and provided by the PDS. One-third of dental professionals reported that they were satisfied with the collaboration they had with RIs. Most of the respondents agreed that personnel from RIs could positively influence interprofessional collaboration by having sufficient knowledge of drug users statutory rights to free of charge dental treatment, as well as by close follow-up and motivation of patients before dental treatment.

**Conclusions:**

Dental professionals perceived the management of drug users as demanding due to dental fear, difficulties in coping with appointments, poor compliance to preventive measures, and disagreement between dental treatment defined as necessary and drug users’ expectations. Attitudes and experiences related to dental treatment of drug users were significantly associated with background characteristics of clinicians. Organizational barriers regarding leadership, accessibility, and collaborative routines, as well as lack of interprofessional communication, suggest current models of health care delivery to drug users need reviewing.

## Background

Drug users represent a group with multiple health challenges, including serious oral health problems, high unmet treatment needs, and reduced oral health-related quality of life [1–4]. Prevention and treatment of oral diseases among drug users might facilitate their rehabilitation and recovery from substance use disorder, both in terms of elimination of pain and discomfort, but also in enhancing their appearance and self-esteem [5–7]. It has been shown that the problems drug users encounter with health services are often associated with barriers related to both structural (service delivery) and individual factors (patient factors) [8].

In Norway, the Public Dental Service (PDS) run by the 18 county councils using salaried personnel offers free treatment for children and adolescents up to 19 years of age. Also, mentally disabled adults, the elderly in nursing homes, persons having nursing services at home, and refugees receive free care from the PDS. Other non-prioritized adults pay for treatment, but their share of all the patients in the PDS is low. Since 2005, adults with substance use disorder that are in rehabilitation institutions under specialist treatment, medication-assisted rehabilitation programs, or under municipal care due to their drug dependence also have free of charge necessary dental care in the PDS. Besides emergency treatment, necessary dental care usually consists of examinations, preventive and selective restorative treatments to assure satisfactory chewing function, and being able to communicate and socialize without problems caused by the teeth [9].

Information on the use of dental services by drug users is scarce in Norway, and the exact numbers of drug users that are eligible for free dental services in the PDS are unknown. In 2013, 7371 drug users were examined and treated in the PDS, and the expenses of their dental treatment were approximately NOK 60 million [10]. There are substantial variations between the counties on how the dental services for drug users are delivered, but no statistical data are collected [11].

In general, drug users are likely to exhibit low adherence to medical advice and treatment regimens [12, 13], irregular dental attendance patterns, dental fear, and behavioral management problems [1, 2, 11, 14]. They are considered to be one of the patient groups dentists felt least comfortable to treat [15], and drug users themselves have reported negative experiences with the dental care system or non-welcoming attitudes from dental professionals in Norway [16]. It is important to be aware of clinicians’ experiences in providing oral health care and to develop appropriate dental services for drug users.

Because of their many health problems, drug users are often in contact with several health care services. In general health care, increased effectiveness, better quality of care, and reduction of health disparities have been associated with successful inter-professional collaboration [17–19]. Collaboration between dental practitioners and non-dental primary care providers has been pointed out as a potential factor to improve oral health care among patients with mental health problems [20]. However, the dental health care delivery system in Norway is separated from the general health care system, hence collaboration can be challenging [21, 22]. It is, therefore, important to assess barriers and facilitators for interprofessional collaboration on organizational, professional, and service user levels.

The present study aimed to assess dental professionals’ attitudes and experiences regarding the dental treatment of drug users in the PDS and to identify barriers and facilitators of interprofessional collaboration between the PDS and rehabilitation institutions.

## Methods

To explore the topic of dentist and dental hygienist perceptions about patients who suffer from substance use disorder, a questionnaire consisting of a series of items was developed. The questionnaire items were generated using informal meetings with several dentists and dental hygienists from PDS units in Norway. The questionnaire comprised two main sections: background characteristics of clinicians and attitudes and experiences to dental treatment of drug users at PDS, including experiences with interprofessional collaboration. The questionnaire was tested for face validity among 3 dentists and 2 dental hygienists to ensure respondents comprehension of the questions and length of the questionnaire. Content validity was also assessed with the same clinicians to determine the question accuracy of capturing the intended research questions.

As a result a semi-quantitative questionnaire was developed, with both close and open-ended items. The items in the questionnaire were: (1) experiences and practice of treatment of this patient group (Longer appointments for drug users (yes/no); Need extra time for: -Information; −Communication;-Motivational interview; −Treatment planning; −Preventive procedures; −Treatment; −Infection control), (2) comprehensions of drug users as patients (Drug users: -have dental fear more often compared to other patient groups; −often have missing appointments; −have poor compliance with oral hygiene advice; −have poor general hygiene; −have increased the risk for transmissible diseases), (3) if the PDS organization facilitates treatment of drug users (It is accepted to use more time for examination of drug users; It is accepted to use more time for treatment of drug users; There are economic barriers in treating drug users; It is important to maintain free of charge dental treatment for drug users); (4) perceived competence in treatment of drug users and experiences (I feel anxious to treat drug users; I feel competent in communication with drug users; I feel competent in the treatment of drug users; I need more knowledge in the dental treatment of this group) and (5) dental clinicians’ opinions on interprofessional collaboration between the PDS and rehabilitation institutions (RIs) (“What do you think are the good things with having collaboration with RIs?” and “In your opinion, which barriers for collaboration do exist?”).

Also, some background data was collected from the dentists and dental hygienists doing the work (gender, country of graduation: Norway versus other countries), as well as undergraduate and postgraduate education in treating drug users. Years of clinical experience were dichotomized into 10 years or less and more than 10 years. Clinical experience with drug users was assessed by the number of drug users treated per month (1–4 and 5 or more). Responses to questions about attitudes and experiences (questionnaire items 2–4) were scored on a five-point Likert scale with the alternatives “strongly agree”, “agree”, “neither agree nor disagree”, “disagree”, and “strongly disagree”. For statistical analyses, the responses were dichotomized into positive attitude (“strongly agree”, “agree”) and negative/neutral attitude (“neither agree nor disagree”, “disagree” and “strongly disagree”).

Questions about dental clinicians’ opinions on interprofessional collaboration between the PDS and RIs were formulated as open questions allowing the respondents to answer the questions in their own words and thus to gather more in-depth answers. Responses were analyzed by descriptive analysis [23, 24] and grouped into barriers and facilitators for interprofessional collaboration.

The study was approved by the Norwegian Centre for Research Data (application 50,791). All participants received written information about the study and consented to participate by submitting the questionnaire.

The questionnaire was electronically distributed to all dentists and dental hygienists (*n* = 194) in the PDS in three counties in the Eastern Norway (Hedmark, Østfold, and Oppland). Data collection was performed during January–March in 2017 using Questback Essentials electronic research platform. For non-responders, three reminders were sent. Data were processed and analyzed using the SPSS statistical program package (IBM SPSS 24.0, SPSS Inc., Chicago, IL, USA). Frequency distributions were used for descriptive statistics, and a Chi-squared test was used to compare differences in responses between groups. Single statements on binary responses describing clinicians’ attitudes and experiences to treatment of drug users (questionnaire items 2–4) were selected as dependent variables and analyzed using the binary logistic regression model. To assess the association between the number of drug users treated per month and the binary outcomes, the binary logistic regression models were adjusted for gender, the profession of the clinicians (dental hygienist versus dentist), country of education and years of clinical experience All models were fitted using StataSE 16 and the significance level was set at α = 0.05.

## Results

The response rate was 65% for dentists (97 of the 145 dentists responded) and 90% for dental hygienists (44 of the 49 dental hygienists responded). In total, 141 (73%) dentists and dental hygienists responded. Five dental hygienists and one dentist were excluded from further analyses because they had not treated drug users. Thus, the final sample for analyses comprised of 135 clinicians, 96 dentists, and 39 dental hygienists. The majority of respondents were female (74%).

While all dental hygienists were graduated from Norway, 31% of responding dentists were educated outside of Norway. Forty-one percent of dentists educated in Norway, 38% of dentists educated abroad and 68% of the dental hygienists had had an undergraduate education in dental management of drug users. The majority of respondents (72%), both dentists (71%) and dental hygienists (74%), had had postgraduate education in the treatment of drug users at their working place, 62% of them had done so during the last year. Among all respondents, 56% of dentists and 62% of dental hygienists reported having over 10 years of clinical experience.

Most respondents (71%) did not treat drug users very often. A third of the dentists (35%) and 10% of the dental hygienists had treated five or more drug users per month (*p* <  0.05). There was a tendency that clinicians with more clinical experience treated drug users more often (not statistically significant).

Seventy-five percent of respondents reported that they set up longer appointments for drug users than for other patients. Dental hygienists more often than dentists reported that drug users required more time for communication (93% versus 70%, *p* = 0.018), motivational interviews (80% versus 37%, *p* = 0.001), and preventive procedures (90% versus 41%, *p* = 0.001), while dentists more often than dental hygienists reported a need for additional time for treatment planning (69% versus 27%, *p* = 0.001) and treatment procedures (73% versus 23%, p = 0.001).

The majority of the respondents stated that drug users often had missing appointments and poor compliance with oral hygiene advice, in addition to poor general hygiene and increased risk for transmissible diseases (Table 1). The respondents also experienced that drug users had dental fear more often compared to other patient groups; however, those who treated drug users more often reported less dental fear.

**Table 1:** Distribution (%) of Norwegian dental professionals (*n* = 133) with positive responses (agree or strongly agree) to the questions regarding experiences of drug users as dental patients in relation to the number of drug users treated per month

Drug users	Total (*n* = 133)%	Number of drug users treated per month	
		Less than 5 (*n* = 96) %	5 or more (*n* = 37) %
-have dental fear more often compared to other patient groups	85	90	76*
-often have missing appointments	91	93	89
-have poor compliance with oral hygiene advice	76	72	86
-have poor general hygiene	74	72	78
-have increased the risk for transmissible diseases	84	82	89

*indicates statistically significant differences (Chi-squared test, *p* <  0.05)

The majority of dentist and dental hygienists reported that there is acceptance for using more time for examination and treatment of drug users in their PDS clinics (Table 2). Thirty-nine percent of the respondents experienced economic barriers to providing treatment and a significantly higher proportion of clinicians treating drug users often reported experiencing economic barriers in treating drug users.. About 80% of the respondents felt that it was important to maintain the free of charge necessary dental treatment for this patient group (Table 2).

**Table 2:** Distribution (%) of Norwegian dental professionals (n = 133) with positive responses (agree or strongly agree) to the questions regarding experiences of organizational facilitation of the treatment of drug users in relation to the number of drug users treated per month

	Total(n = 133) %	Number of drug users treated per month	
		Less than 5 (n = 96) %	5 or more (n = 37) %
It is accepted to use more time for examination of drug users	68	72	60
It is accepted to use more time for treatment of drug users	73	72	76
There are economic barriers in treating drug users	39	32	56*
It is important to maintain free of charge dental treatment for drug users	81	83	76

*indicates statistically significant differences (Chi-squared test, *p* <  0.05)

As shown in Table 3, more than 90% of the respondents felt competent in the clinical treatment of this group. A slightly lower proportion (84%) felt competent in communication with them and 71% considered dental treatment of drug users as professionally satisfying. Still, 17% of respondents felt anxious to treat drug users, and 80% felt they needed more knowledge in dental treatment of this group. There were no significant differences in relation to the number of drug users treated per month.

**Table 3:** Distribution (%) of Norwegian dental professionals (n = 133) with positive responses (agree or strongly agree) to the questions regarding perceived competence in treating drug users in relation to the number of drug users treated per month

	Total(*n* = 132) %	Number of drug users treated per month	
		Less than 5 (n = 96) %	5 or more (*n* = 36) %
I feel anxious to treat drug users	17	18	14
I feel competent in communication with drug users	84	84	84
I feel competent in the treatment of drug users	93	93	95
I need more knowledge in the dental treatment of this group	80	78	86

Tables 4 and 5 show the results of the multivariable analyses exploring associations between background variables and single significant attitudinal statements. Gender, number of drug users treated per month, the profession of the clinicians (dental hygienist versus dentist), country of education and years of clinical experience were associated with professionals’ attitudes and experiences regarding dental treatment of drug users. Males were less likely to perceive drug users having dental fear (OR 0.17, CI 0.05, 0.54), less supportive to free of charge dental treatment (OR 0.27, CI 0.09, 0.75) and were less likely in need for more knowledge on treatment of drug users (OR 0.20, CI 0.07, 0.57).

**Table 4:** Multivariable logistic regression analysis of practitioners’ comprehensions of drug users as patients and explanatory variables (gender, number of drug users treated per month, profession, country of education and years of clinical experience (*n* = 133). Odds ratios (OR), 95% confidence interval (CI) and *P*-values

Explanatory variables	Dental fear^a^		Missing appointments^b^		Risk of transmissible diseases^c^	
	OR (95% CI)	*P*-value	OR (95% CI)	*P*-value	OR (95% CI)	*P*-value
Gender (ref: Female)						
Male	**0.17 (0.05, 0.54)**	**0.01**	0.38 (0.09, 1.65)	0.20	0.59 (0.18, 1.89)	0.38
Drug user treated per month (ref: < 5)						
5 or more	0.50 (0.16, 1.53)	0.22	0.59 (0.13, 2.62)	0.49	2.13 (0.57, 7.97)	0.26
Profession (ref:Dental hygienist)						
Dentist	0.93 (0.21, 4.13)	0.92	0.96 (0.20, 4.61)	0.95	2.88 (0.80, 10.41)	0.11
Educated in Norway (ref: Yes)						
No	1.46 (0.40, 5.35)	0.57	5.20 (0.53, 51.11)	0.16	**0.16 (0.05, 0.56)**	**< 0.01**
Clinical experience (ref: > 10 years)						
10 years or less	2.01 (0.63, 6.41)	0.24	**0.20 (0.05, 0.85)**	**0.03**	1.46 (0.53, 4.02)	0.46

^a^ Drug users have dental fear more often compared to other patient groups

^b^ Drug users often have missing appointments

^c^ Drug users have increased the risk for transmissible diseases

**Table 5:** Multivariable logistic regression analysis of experiences of organizational facilitation and perceived competence and explanatory variables (gender, number of drug users treated per month, profession, country of education and years of clinical experience (n = 133). Odds ratios (OR), 95% confidence interval (CI) and P-values

Explanatory variables	More time for treatment^a^		Economic barriers for treatment^b^		Free of charge dental treatment^c^		Need for more knowledge^d^	
	OR (95% CI)	*P*-value	OR (95% CI)	*P*-value	OR (95% CI)	*P*-value	OR (95% CI)	*P*-value
Gender (ref: Female)								
Male	0.72 (0.29, 1.83)	0.50	1.86 (0.78, 4.42)	0.16	**0.27 (0.09, 0.75)**	**0.01**	**0.20 (0.07, 0.57)**	**< 0.01**
Drug user treated per month (ref: < 5)								
5 or more	1.50 (0.58, 3.87)	0.40	**2.26 (1.00, 5.24)**	**0.05**	0.68 (0.24, 1.91)	0.46	2.75 (0.82, 9.18)	0.10
Profession (ref:Dental hygienist)								
Dentist	0.87 (0.32, 2.37)	0.78	1.26 (0.50, 3.20)	0.62	2.51 (0.79, 7.97)	0.12	1.52 (0.48, 4.83)	0.48
Educated in Norway (ref: Yes)								
No	**0.36 (0.14, 0.94)**	**0.04**	1.61 (0.63, 4.11)	0.32	0.74 (0.23, 2.34)	0.60	0.56 (0.18, 1.76)	0.32
Clinical experience (ref: > 10 years)								
10 years or less	1.10 (0.49, 2.46)	0.82	0.95 (0.45, 2.00)	0.88	1.29 (0.50, 3.31)	0.60	1.82 (0.68, 4.82)	0.23

^a^ It is accepted to use more time for treatment of drug users

^b^ There are economic barriers in treating drug users

^c^ It is important to maintain free of charge dental treatment for drug users

^d^ I need more knowledge in the dental treatment of this group

Less experienced clinicians were less likely to experience drug users having missing appointments (OR 0.20, CI 0.05, 0.85). Clinicians educated outside of Norway were less likely to perceive drug users having increased the risk for transmissible diseases (OR 0.16, CI 0.05, 0.56) as well as to accept to use more time for treatment of drug users (OR 0.36 CI 0.14, 0.94). The odds of experiencing economic barriers were marginally significantly higher among clinicians treating 5 or more drug users per month (OR 2.26, CI 1.00, 5.24).

Forty percent of the respondents reported that they had daily, weekly, or monthly communication with employees at rehabilitation institutions. A third of the dentists (33%) and 21% of the dental hygienists responded that they were satisfied with the collaboration they had with the RIs.

The respondents in this study answered that the interprofessional collaboration was facilitated when the RI staff was easily available for communication (e.g., by telephone), felt responsible for assisting drug users in coping with dental appointments, and when they had knowledge of the treatment included and not included in the free dental care provided in the PDS (Table 6). Dental personnel emphasized the importance of having the necessary knowledge about patients’ medications and general health status. Several respondents pointed out that the fact that “*patients are driven and followed by the RI staff to the dental clinic*” results in fewer drop-outs and in a higher quality of dental treatment for drug users. At the same time, lack of communication and contact between the two service sectors was seen as a barrier for collaboration (Table 6).

**Table 6:** Barriers and facilitators for interprofessional collaboration seen from dental professionals’ perspectives

Theme	Barriers	Facilitators
Professionals in the RI^a^s	• Lack of communication • Difficult to communicate • A large number of employees • A large number of patients • Long waiting time for dental treatment	• Good communication • Easy to communicate by phone • Holding appointments • Informing about appointments changes
	• Lack of patient follow-up by RI^a^ personnel • Lack of information about patients before dental appointment/treatment	• Good patient follow-up by RI^a^ personnel • Good information about patients ahead of a dental appointment • RI^a^ personnel drive patients to a dental clinic
	• Lack of knowledge about drug users and their statutory rights	• Good knowledge about drug users and their statutory rights
Patients (drug users)	• Lack of motivation • Negative attitudes • Appointments drop-outs	• RI^a^ personnel motivate patients
	• Lack of knowledge about statutory rights • Lack of knowledge about treatments limitations • A high expectation of dental treatment	• RI^a^ personnel informs patients about their statutory rights
Organizational context		• Leadership support
	• Lack of meeting arenas	• Regular collaboration meetings

^a^RI: Rehabilitation institution

Motivated patients were also a facilitator for successful collaboration between dental clinics and rehabilitation institutions. The respondents appreciated the support from RI staff, highlighting the importance of dental care but also not rising unrealistic high expectations on dental treatment. “*Patients with high expectations and no understanding of principles of dental treatment*” were seen as an obvious barrier for interprofessional collaboration (Table 6).

## Discussion

In our study, we show that the management of drug users as patients in PDS is demanding due to their dental fear and difficulties in coping with appointments and poor compliance with preventive measures. Although the great majority of the responding dentists and dental hygienists (93%) felt competent in the dental treatment of drug users under rehabilitation, 80% of the respondents reported that they needed more knowledge of the management of this patient group, and almost one in five (17%) felt anxious to treat them. Furthermore, our results show that drug users’ behavior could be challenging, and some dentists and dental hygienists might have been fearful of HIV or other infections the patients could carry. This was in accordance with findings several other studies from another Norwegian study [25] and supported by Helvig et al. [6], who reported that approximately half of the drug users completed the planned courses of dental treatment and about 20% of the dental appointments were dropouts [6, 25].

Interprofessional collaboration has been identified as a critical factor in delivering quality health services and reducing health disparities in vulnerable patient groups [26, 27].

In recent years, the Norwegian government has been strengthening the interprofessional collaboration between health care institutions [18] and reducing social inequalities for vulnerable patient groups However in the present study, only 33% of the dentists and 21% of the dental hygienists responded that they were satisfied with the collaboration they had with the RIs. The qualitative part of this study revealed that in spite of the new initiatives, the collaboration between professionals in the PDS and the RIs was rather limited. In Norway, the PDS is organized at the county level, while institutions involved in the rehabilitation of drug users are organized at the municipal or governmental level. This difference in organizational structure, together with differences in management levels and lack of communication, has been previously reported to hamper collaboration between different healthcare providers [28, 29].

The results generated from the qualitative part of the questionnaire are in line with those of studies showing that factors such as frequent communication, professionals knowledge, and available resources are important for successful interprofessional collaboration. These results suggest that it would be possible to reduce drug users dropouts from the dental appointments by closer follow-up of the patients at the rehabilitation institution, which includes driving them to dental clinics, reminding them about appointments, and motivating them for dental treatment. Furthermore, access to relevant information before dental treatment about patients’ health conditions, medication use, and dental phobia was reported as very useful information for dental clinicians and seems to facilitate the collaboration between the institutions. Increased knowledge and understanding of drug dependence have been shown to lead to better and more effective patient management [30], and knowledge at both patient and service levels was also highlighted as an important factor in facilitating good interprofessional collaboration.

It was obvious that drug users’ dental attendance behaviour could be challenging despite them receiving rehabilitation treatment. In the open questions, the respondents pointed out that drug users often had higher expectations for dental treatment than what could be defined as necessary care and provided by PDS. This certainly could be a source of disappointment for many patients. In the PDS, only dental care considered as necessary is available free of charge for drug users under rehabilitation. According to the clinical guideline issued by Norwegian Ministry of Health [9], necessary dental care consists of examinations, preventive, selective restorative and prosthetic treatments to assure satisfactory chewing function, communication, and socialization without problems caused by the teeth. This means that the clinical treatment measures provided in the PDS, as such, might not have been technically complicated.

From a health policy point of view, free of charge necessary dental care for drug users under rehabilitation can be considered to be a beneficial (and necessary as the respondents pointed out) support to the ongoing rehabilitation program. On the other hand, a disagreement between dental treatment defined as necessary by PDS and drug users’ expectations might be a challenge to providing care. More than half (56%) of dental professionals who treated drug users often had experienced economic barriers related to treatment provision. One possible explanation for this could be budgetary limitations in the PDS. It has to be noticed that emergency services and dental care for active drug users – in the same way as most other adults in Norway – are provided in the private sector and paid by the patients out-of-pocket. There is, however, little information on how the provision of dental care for drug users works in the private sector.

The present study was a questionnaire study, and the respondents self-selected to complete the survey. Thus, selection bias related to personal interests of clinicians as well as recall bias cannot be ruled out. To the best of our knowledge, the present study is the first in Norway to investigate dental practitioners’ experiences with interprofessional collaboration in treating drug users. The response rate was good, and the results can be considered representative of dentists and hygienists in the region.

In summary, as treatment of this patient group seemed to require a considerable amount of further education, it can be questioned whether it would be more feasible and profitable for the PDS organization and better for the patients to concentrate this kind of rare treatment to certain teams in the individual PDS units, instead of demanding special knowledge, preparedness to make inter-professional contacts, as well as other niche demands of all employees. Earlier studies have indicated that experience and expertise are important predictors of health care quality for drug users [31]. Most dentists and dental hygienists in this study had treated drug users, but the numbers of patients treated were low. Greater experience of treating drug users might help clinicians develop the understanding and skills that make it easier to treat such patients more effectively [31]. This must be true also for dental care.

## Conclusions

Dental professionals perceived the management of drug users as demanding due to dental fear, difficulties in coping with appointments, poor compliance to preventive measures, and disagreement between dental treatment defined as necessary and drug users’ expectations. Attitudes and experiences with dental treatment of drug users were significantly associated with background characteristics of professionals. The organizational barriers regarding leadership, accessibility, and collaborative routines reported in the present study, as well as lack of interprofessional communication, suggest current models of health care delivery to drug users need reviewing.

## Data Availability

The dataset used in the current study is available from the corresponding author on reasonable request.

## Abbreviations

PDS: Public Dental Service
RIs: Rehabilitation institutions
OR: Odds ratio
CI: Confidence interval
HIV: Human immunodeficiency virus
